# A Proof-of-Concept of a 2-Hours Direct Antimicrobial Susceptibility Test from Inoculated Urine Samples

**DOI:** 10.3390/microorganisms14030711

**Published:** 2026-03-22

**Authors:** Mariana Sousa-Pinheiro, Inês Martins-Oliveira, David Abreu, Rosário Gomes, Ana Silva-Dias, Patrícia Poeta, Cidália Pina-Vaz, António José Soares

**Affiliations:** 1 RISE-Health, Faculty of Medicine, University of Porto, 4200-319 Porto, Portugal; marianapinheiromp11@gmail.com (M.S.-P.); ajasoares@med.up.pt (A.J.S.); 2 Department of Veterinary Sciences, University of Trás-os-Montes and Alto Douro (UTAD), 5000-801 Vila Real, Portugal; 3 FASTinov, SA, 4200-135 Porto, Portugal; 4 Microbiology and Antibiotic Resistance Team (MicroART), Department of Veterinary Sciences, University of Trás-os-Montes and Alto Douro (UTAD), 5000-801 Vila Real, Portugal; ppoeta@utad.pt; 5 CECAV-Veterinary and Animal Research Centre, University of Trás-os-Montes and Alto Douro (UTAD), 5000-801 Vila Real, Portugal; 6 Associate Laboratory for Animal and Veterinary Sciences (AL4AnimalS), University of Trás-os-Montes and Alto Douro (UTAD), 5000-801 Vila Real, Portugal

**Keywords:** urine, antimicrobial resistance, rapid antimicrobial susceptibility testing, urinary tract infections, flow cytometry

## Abstract

Urinary tract infections (UTIs) are the most frequent infections in hospitalized and outpatient settings, where *Escherichia coli* is the predominant pathogen. Conventional diagnostic and antimicrobial susceptibility testing (AST) methods are time-consuming, often requiring 48 h, leading to empirical antibiotic therapy and contributing to antimicrobial resistance (AMR). FASTinov^®^ developed a rapid phenotypic method that enables AST directly from urine samples within two hours using flow cytometry. In this study, 154 inoculated urine samples were analyzed to evaluate the performance of two diagnostic panels: FASTgramneg for Gram-negative bacteria and FASTgrampos for Gram-positive bacteria. Data analysis was performed using bioFAST^®^ software (version 3.0), providing results in accordance with EUCAST guidelines. The FASTgramneg panel allows detection of resistance mechanisms, including extended-spectrum β-lactamases (ESBLs), and screening of AmpC β-lactamases and carbapenemases; the FASTgrampos panel additionally determines the minimal inhibitory concentration (MIC) of vancomycin for *Staphylococcus aureus*. Overall agreement with conventional AST methods was 97.5% for Gram-negative bacteria and 95.0% for Gram-positive bacteria. All resistance mechanisms were correctly identified with no false positives. The essential agreement for vancomycin’s MIC was 95.2%, with a BIAS of +14.3%. Reproducibility was 99.5% for FASTgramneg and 95.0% for FASTgrampos. These results demonstrate that the FASTinov^®^ kit significantly reduces turnaround time while maintaining high accuracy, supporting improved UTI management and antimicrobial stewardship.

## 1. Introduction

Urinary tract infections (UTIs) encompass a range of infectious syndromes affecting the urinary tract, from the urethra to the kidneys, afflicting almost 400 million people each year worldwide [1]. They are among the most common bacterial infections in both hospital and outpatient settings [2], with an estimated 50–60% of women experiencing at least one UTI during their lifetime and the infection risk increasing with age [3,4,5]. Some risk factors include urinary retention, frequent sexual intercourse, the use of spermicides and anatomical abnormalities (i.e., prostatic gland enlargement and vulvovaginal atrophy) [6]. *Escherichia coli* is the most frequently isolated pathogen, encompassing at least 80% of community-contracted infections and 65% of hospital-contracted ones, but many others, such as *Klebsiella pneumoniae*, *Proteus* spp., *Pseudomonas* spp., and Gram-positive cocci, namely *Staphylococcus* spp. and *Enterococcus* spp., are also implicated [6,7]. Current recommendations from major guidelines (e.g., IDSA and the European Association of Urology) highlight that urine culture is recommended in cases of suspected complicated UTIs, pyelonephritis, treatment failure, recurrent infections, and pregnancy and in patients at a higher risk for antimicrobial resistance. Actually, diagnosis primarily relies on urine culture [1,8], which typically requires a minimum of 24 h just to isolate and identify the microorganism and 48 h to yield AST results. These limitations delay the initiation of targeted therapy, and as such, an empirical one is often initiated without definitive microbial identification (ID) or susceptibility data. In the context of uncomplicated sporadic UTIs in otherwise healthy women, rapid diagnostic tools may have a limited impact as empirical therapy remains highly effective. However, their utility may become clinically relevant in women with individual risk factors for antimicrobial resistance—such as recent antibiotic exposure, prior infection with resistant organisms, recent hospitalization, or international travel—where the likelihood of empirical treatment failure is higher. In contrast, in patient populations with a greater baseline prevalence of resistant microorganisms—such as those with recurrent UTIs, prior colonization by multidrug-resistant organisms, urinary tract abnormalities, indwelling catheters, or other forms of complicated UTIs—the potential value of rapid diagnostic tools may be more substantial. In these higher-risk settings, early pathogen identification and resistance profiling could meaningfully support timely optimization of antimicrobial therapy, antimicrobial stewardship efforts, and improved clinical outcomes. Distinguishing between these clinical scenarios is essential when assessing the potential role and impact of novel rapid diagnostic strategies. This approach can contribute to therapeutic failure and drug toxicity and promote antimicrobial resistance (AMR) due to inappropriate antibiotic use [3]. Despite advances in diagnosis and treatment, UTIs continue to be associated with high morbidity and mortality rates [2]. Therefore, there is a pressing need for rapid, point-of-care AST platforms capable of delivering accurate results within hours, namely in urine samples. The 2023 World Health Organization (WHO) antibacterial pipeline report reflects a shift toward narrow-spectrum agents, which further necessitate the development and integration of rapid diagnostic tools to ensure appropriate, patient-specific use [9]. AMR is a real threat to global health, with approximately 5 million directly and indirectly associated deaths in 2019 [10]. AST plays a pivotal role in guiding effective therapy by determining the susceptibility of pathogens to specific antimicrobials, which facilitates tailored treatment, helps avoid the unnecessary use of broad-spectrum antibiotics, and supports the stewardship efforts essential to curbing the rise in drug-resistant organisms. Rapid AST is crucial, especially in the context of rising resistance such as that associated with carbapenem-resistant Enterobacterales. A recent rapid risk assessment by the European Centre for Disease Prevention and Control (ECDC) underscores the need for prompt diagnostics, including AST, to optimize the use of novel antibiotics [11].

Flow cytometry represents a major advance in microbiological diagnosis. It allows the study of individual cells in terms of their morphology, structure and functional aspects. This technology has the capability of determining susceptibility phenotypes and, at the same time, can characterize some mechanisms of resistance; as it is not growth-dependent, it could provide rapid AST reports [12].

FASTinov^®^, a spin-off of Porto University, has been using a disruptive technology based on flow cytometry analysis, providing a rapid phenotypic antimicrobial susceptibility test that compares cells treated with antibiotics for short periods such as 1 h with non-treated cells. The detection of cellular lesions induced by different antibiotics was performed with the addition of fluorescent probes [12]. An artificial algorithm was used to perform a multiparametric analysis and provide a report in less than 2 h. Its use on positive blood cultures showed excellent results, avoiding the subculture steps and reducing the AST time from the expected 48 h to approximately 2 h [13]. Using urine samples screened as positive for Gram-negative bacilli or Gram-Positive cocci by systems like Syxmex^®^ [14], it would be possible to apply the same approach to urine, similar to the blood culture approach. Negative or contaminated samples should be excluded. A rapid susceptibility protocol using colistin was already performed directly on urine samples [15]. In this paper, inoculated urine samples with well-characterized bacteria were validated, and the results were compared with the expected ones.

## 2. Materials and Methods

### 2.1. Urine Inoculation

Urine samples were collected from healthy volunteers and inoculated with selected bacteria from the FASTinov culture collection following the protocol described by Roos et al. [16] and incubated overnight. The bacterial strains used for inoculation are described in Table 1. It included several control stains, namely *Staphylococcus aureus* ATCC 29213, *S. aureus* ATCC 43300, *S. aureus* ATCC 700221, and *S. epidermidis* ATCC 35984 for Gram-positive bacteria and *Escherichia coli* ATCC 8739, *E. coli* ATCC 25922, *E. coli* ATCC 35218, *E. coli* BAA 2452, *Klebsiella pneumoniae* ATCC 13443, *K. pneumoniae* ATCC 700603, *K. pneumoniae* BAA 1705, *K. pneumoniae* BAA 1706, *Serratia marcescens* ATCC 14756, and *Pseudomonas aeruginosa* 27853 for Gram-negative bacteria. In total, 154 samples were inoculated and incubated overnight. All the strains were identified by MALDI, and susceptibility was determined by microdilution and/or disk diffusion according to EUCAST recommendations, with strains classified as susceptible (S), resistant (R) susceptible with increase exposure (I).

### 2.2. Sample Concentration

Eight to ten mL of each inoculated urine sample was centrifuged at 4000 rpm for 10 min. The supernatant was discarded, and the pellet was resuspended in 1 mL of sterile, deionized water.

### 2.3. FASTinov Kit Inoculation

Based on the microbial ID, two kits (FASTgramneg (for Gram-Negative bacteria) and FASTgrampos (for Gram-Positive bacteria)) could be used. Each kit consists of a 96-well microplate panel containing several antibiotics (see Table 2 and Table 3) at breakpoint concentrations according to EUCAST, as well as an optimized fluorescent probe to detect bacterial cell damage. One fluorescent probe was used on the FASTgramneg kit, while 2 were used on the FASTgrampos kit. Each drug was compared with the respective controls (cells without drug exposure and stained with the probe). The FASTgramneg kit was inoculated with 100 µL of a bacterial suspension in the first 5 rows (A–E), and the FASTgrampos kit was inoculated in the first 3 (A–C) according to the kit manufacturer’s instructions. The panels were then incubated for 1 h at 35 ± 1 °C with shaking (750 rpm) and analyzed in a flow cytometer.

### 2.4. Flow Cytometry Analysis

A minimum of 30,000 cells per well were analyzed using a DxFlex Flow Cytometer (Beckman Coulter^®^, Brea, CA, USA). Treated samples were compared with untreated control samples through multiparametric analysis. To assess the AST results, a proprietary artificial intelligence- and machine learning-based software (bioFAST^®^ version 3.0) using a large dataset of known bacterial phenotypes was used, This assessment was done automatically immediately after the flow cytometry analysis. The susceptibility results were recorded according to the EUCAST criteria; a report of either susceptible (S), intermediate (meaning susceptible with increased exposure) (I), or resistant (R) to each drug was produced. The FASTinov^®^ FASTgramneg test is also able to identify some resistance mechanisms, such as the detection of ESBL, which was determined by screening for AmpC and carbapenemases according to the EUCAST protocol; the FASTinov^®^ FASTgrampos test provides MIC values for vancomycin in case of *S. aureus.*

### 2.5. Reproducibility Assessment

At least ten samples with the MIC on scale for each drug were tested in triplicate, and reproducibility was assessed by comparing the categorical results (susceptible/resistant) obtained across the replicates and calculating the percentage of agreement between runs.

### 2.6. Statistical Analysis

Sensitivity, specificity, and overall accuracy were calculated to evaluate the performance of the method when compared to the reference method [17]. Discrepant results were repeated using FASTinov and the reference method at the same time, and the second experiment was considered.

## 3. Results

**Figure 1:**
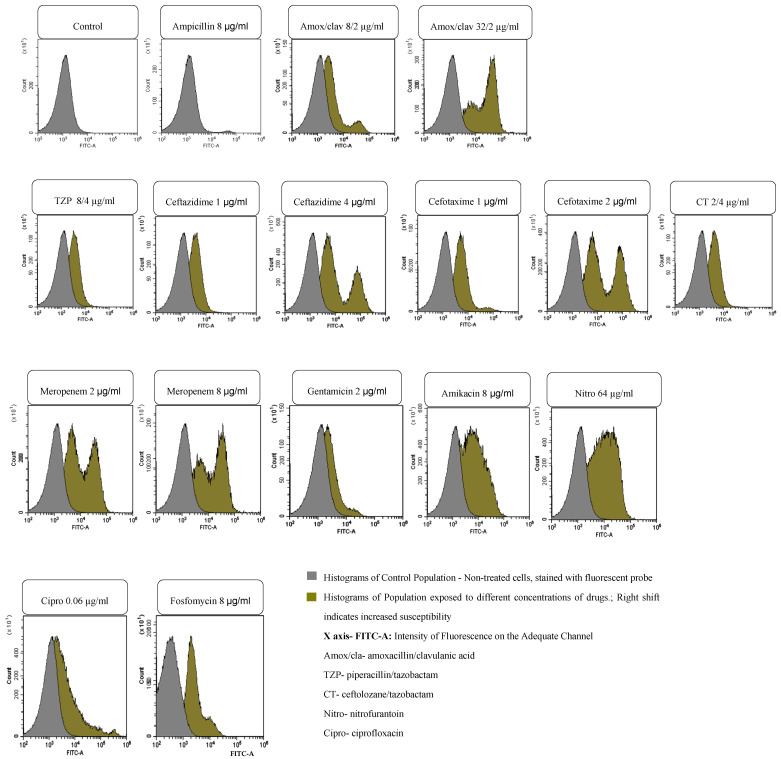
Example of the analysis of an *E. coli* strain (ATCC 25922) susceptible to all drugs except ampicillin by flow cytometry.

**Figure 2:**
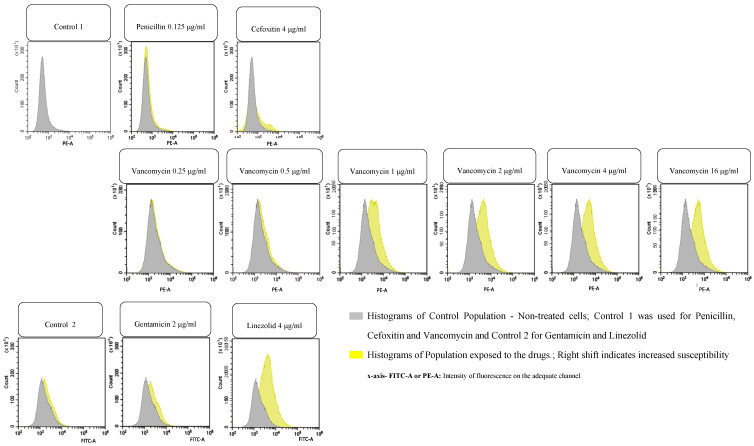
Example of the analysis of an *S. aureus* strain (ATCC 29213) resistant to penicillin, cefoxitin (MRSA), and gentamicin but susceptible to vancomycin (MIC = 1 µg/mL) and linezolid by flow cytometry.

Various strains belonging to different genera, eight Gram-negative and two Gram-positive (Table 1), and possessing different antimicrobial phenotype patterns, including several resistant strains, 191 Gram-negative and 102 Gram-positive (Table 2 and Table 3), were obtained as expected. FASTgramneg showed a sensitivity of 96.3% and a specificity of 97.8%, with an accuracy of 97.5%; fifteen strains were ESBL-positive, with four AmpC producers and eight carbapenemase producers (four KPC, two metallo-carbapenemases and two OXA-like 48). Although at a reduced number, they were correctly detected with no false positives. Regarding FASTgrampos, the sensitivity was 92.2%, while the specificity was 96.3%, with an accuracy of 95.1%. The lowest CA value was found with gentamicin, with five VMR, which is high, but the number of resistant strains was low and needs to be increased. The minimum inhibitory concentration (MIC) to vancomycin determined by microdilution was between 0.25 and 1 µg/mL for all the *S. aureus* strains tested; the essential agreement (EA) of the FASTinov test was 95.2% (two strains showed MIC = 2 µg/mL, with one dilution above the reference method). BIAS was +14.3%. The total number of minor errors (mEs) was very low; regarding major errors (MEs), they were present specifically in Gram-positive bacteria when the number of susceptible strains was very low. The total number of very major errors (VMEs) depends a lot on the number of resistant strains that should be increased. Reproducibility was 99.5% on the FASTgramnegative test and 95.0% for FASTgrampos. Regarding flow cytometry, Figure 1 and Figure 2 represent examples of histograms of one Gram-negative strain and one Gram-positive strain. The first image on each figure always represents the control of non-treated cells that should be compared with all the treated suspensions. Overlays of the histograms were performed in order to easily compare the treated population with non-treated populations. A shift in the population to the right means cell lesion. A machine learning algorithm developed by analyzing more than 1000 strains is able to classify their phenotypes based on at least 25 cell features, such as size, complexity, and fluorescence, to compare treated cells with non-treated cells. Note that the intensity of fluorescence of the cells is in logarithmic scale, so small shifts mean a great increase in fluorescence.

## 4. Discussion

UTIs are one of the most frequent clinical problems that could pose a real life-threatening situation, with potentially association with sepsis and dissemination of antimicrobial resistance organisms. This is a public health problem with severe financial implications, particularly in the extended hospitalization time that the patients experience.

The current methods of diagnosis are time-consuming, relying on urine cultures just for the identification of the microorganism, with a minimum wait time of 16–18 h for results. In addition to this time, we must account for AST, which requires a minimum of 24 h for the complete diagnosis and implementation of the correct therapy. The PA-100 AST System developed by Syxmex^®^ has the advantage of being a point-of-care test that can quickly determine the targeted antibiotic treatment in just 45 min; the disadvantages of this study are as follows: only five antibiotics were tested with this method; it was only optimized for five bacterial species specially dedicated to uncomplicated UTIs, and it does not provide an indication about identification or contamination [14]. New rapid phenotypic AST approaches involving advanced technologies including microfluidics, monitoring individual bacterial growth [18], bacterial impedance cytometry [19], dual-enzyme-based technology tests [20], dual-enzyme trigger-enabled cascade technology [21] and metagenomic nanopore sequencing [8] have been used in urine samples. More recently, a novel phenotypic impedance-based Fast AST (iFAST) method measured changes in the electrical phenotype of individual bacteria in response to antibiotic exposure, as described in [22]. There is an emerging need for new technologies that are available and viable in the healthcare market.

As for this study, the FASTgramneg test and the FASTgrampos test, which were previously validated in positive blood cultures, showed excellent results in inoculated urine samples. In addition to the drugs used in blood cultures, results on fosfomycin and nitrofurantoin were included and reported in the FASTgramneg test. After clinical validation, this rapid and accurate alternative to traditional AST methods, with a significant reduction in the time to results, could substantially impact the management of UTIs in clinical practice, potentially avoiding empirical therapy and time-consuming methods, which delay the positive course of treatment. Prior to this study, the FASTcolistin MIC test from FASTinov^®^ was already assessed as a fast and precise method for the detection of resistance to colistin directly in urine with UTIs, with a significant reduction in the time to results to under 2 h. Since colistin is used in critical patients without a lot of therapeutic options, this is a valid alternative to the traditional, time-consuming methods.

One limitation is that the current study was performed as recommended using spiked, non-clinical urine samples. We are now conducting an ongoing clinical study that is essential for pre-market introduction. Nonetheless, we expect problems such as the prevalence of polymicrobial urine. The Sysmex system will be used for screening, and samples with a low number of bacteria and/or alerts for possible contamination will be rejected. Polymicrobial samples will be a question for any phenotypic test that works directly with clinical samples. Clinical samples will be processed using a QUICKprep protocol, yielding a pellet containing viable microorganisms and largely free of debris. This will enable identification by MALDI-TOF and, simultaneously, AST. Rapid AST needs rapid ID as having this information provides clinical value. MALDI-TOF may occasionally identify more than one microorganism or fail to provide an identification; in such cases, AST should not be performed. We did not perform identification in this study because the samples were inoculated with known bacterial strains, but we already know that the resultant pellet from the inoculum preparation can be used with MALDI-Tof for identification (paper in press). The implementation of rapid tests should serve as an opportunity to improve pre-analytical processes—such as proper urine collection and rapid transport to the laboratory—as well as post-analytical processes, to ensure prompt communication of results to the treating physician. An expert discussion should highlight the lab workflow in order to define which urine samples should undergo rapid testing, such as those from transplant patients and children, individuals with urosepsis, those with recurrent UTIs, or others.

## 5. Conclusions

In conclusion, a rapid UTI diagnosis can be obtained using both FASTinov^®^ panels, markedly reducing the turnaround time compared to the conventional method, from 48 to 2 h. These findings highlight the potential of this technology for diagnostic purposes and subsequently establish the therapy for UTIs, thereby speeding up the overall workflow with reliable results while minimizing the turnaround time and the need of empirical treatment.

## Figures and Tables

**Table 1:** Urine inoculated strains used in the evaluation of FASTinov kits.

Gram Negative Strains	Number of Samples	Gram Positive Strains	Number of Samples
*Escherichia coli*	32	*Staphylococcus aureus*	42
*Klebsiella pneumoniae*	24	*Staphylococcus epidermidis*	20
*Pseudomonas aeruginosa*	7	*Enterococcus faecium*	7
*Proteus mirabilis*	6	*Enterococcus faecalis*	6
*Serratia marcescens*	2	*Staphylococcus haemolyticus*	1
*Klebsiella aerogenes*	1	*Staphylococcus saprophyticus*	1
*Klebsiella variicola*	1		
*Klebsiella oxytoca*	1		
*Morganella morganii*	1		
*Enterobacter asburiae*	1		
*Providencia rettgeri*	1		
Total Gram-negative 77		Total Gram-positive 77	

**Table 2:** Agreement between results of FASTgramneg and the reference method.

EUCAST									
RM									
Antimicrobial Agent	n	S	I	ATU	R	CA (%)	mE	ME	VME
Ampicillin	70	23	-	-	47	97.0	-	-	2/44
Amoxicillin–clavulanic acid	69	43	-	-	26	100	-	-	-
Cefotaxime	70	53	1	-	16	98.5	-	1/53	-
Ceftazidime	77	54	7	-	16	93.2	4/77	1/54	-
Cefepime	77	53	6	-	18	98.6		1/53	
Piperacillin–tazobactam	77	59	6	-	12	97.3		1/59	2/12
Ceftolozane–tazobactam	75	66	-	2	7	98.6			1/7
Ceftazidime–avibactam	75	72	-	-	3	100	-	-	-
Meropenem	77	75	-	-	2	94.6	1/77	3/75	
Ciprofloxacin	77	50	7	-	20	97.4		2/50	
Gentamicin	70	54	-	-	16	97.1		2/54	1/16
Amikacin	77	73	-	-	4	98.7	-	1/73	-
Nitrofurantoin (only for *E.coli)*	32	30	-	-	2	100	-	-	-
Fosfomycin (only for *E.coli)*	32	30	-	-	2	96.9	-	-	1
Overall	955	735	27	2	191	97.5	5/955	12/735	7/191

RM—reference method; n—number of strains; S—susceptible; I—susceptible with increased exposure (EUCAST); R—resistant; ATU—Area of technical uncertainty; mE—minor error; ME—major error; VME—very major error; CA—categorical agreement; - means that there were no strains.

**Table 3:** Agreement between results of FASTgrampos and the reference methods.

EUCAST								
RM								
Antimicrobial Agent	n	S	I	R	CA (%)	mE	ME	VME
Ampicillin	13	6	-	7	100%	-	-	-
Cefoxitin (except *S. epidermidis*)	42	33	-	9	95%	-	2/33	-
Gentamicin	59	48	-	11	83%	-	5/54	5/11
High-dose Gentamicin (*only E. faecalis*)	6	6	-	-	100%	-	-	-
Linezolid	73	73	-	-	100%	-	-	-
Oxacillin (only *S. epidermidis*)	20	6	-	14	90%	-	-	2/14
Penicillin	63	2	-	61	95%	-	2/2	1/61
Vancomycin	70	70	-	-	100%	-	-	-
Overall	346	244	-	102	95.0%	-	9/244	8/102

RM—reference method; n—number of strains; S—susceptible; I—susceptible with increased exposure (EUCAST); R—resistant; mE—minor error; ME—major error; VME—very major error; CA—categorical agreement; - means that there were no strains.

## Data Availability

The original contributions presented in this study are included in the article. Further inquiries can be directed to the corresponding author.
